# Astragaloside IV Alleviates H_2_O_2_-Induced Mitochondrial Dysfunction and Inhibits Mitophagy Via PI3K/AKT/mTOR Pathway

**DOI:** 10.1155/cdr/9549175

**Published:** 2025-07-10

**Authors:** Miaomiao Qi, Qiongying Wang, Runmin Sun, Zeyi Cheng, Mingze Li, Xin Fan, Feng Bai, Jing Yu

**Affiliations:** ^1^Department of Cardiology, Lanzhou University Second Hospital, Lanzhou, Gansu Province, China; ^2^Department of Cardiac Surgery, Ruijin Hospital, Shanghai Jiaotong University School of Medicine, Shanghai, China; ^3^The Second Clinical Medical School of Lanzhou University, Lanzhou University, Lanzhou, Gansu Province, China

**Keywords:** Astragaloside IV, mitochondrial dysfunction, mitophagy, oxidative stress, PI3K/AKT/mTOR

## Abstract

Oxidative stress and mitochondrial dysfunction play critical roles in the pathology of cardiovascular diseases. However, the effects of Astragaloside IV (As-IV) on mitochondrial function remain unclear. This study is aimed at evaluating the protective effects and mechanism of As-IV against H_2_O_2_-induced mitochondrial dysfunction in H9c2 cells. H9c2 cells were exposed to 200 *μ*M H_2_O_2_ with or without As-IV. The level of apoptosis and reactive oxygen species (ROS) was measured by flow cytometry. Confocal microscopy and transmission electron microscopy were performed to detect the changes in mitochondrial membrane potential (MMP), mitochondrial morphology, and autophagosome. Mitochondrial dynamics and mitophagy-related proteins were measured by Western blot. The results indicated that As-IV decreased H_2_O_2_-induced apoptosis and ROS generation. Meanwhile, As-IV significantly increased MMP, exerted regulatory effects on mitochondrial dynamics, and ameliorated the damaged mitochondrial morphology in H_2_O_2_-injured cardiomyocytes. Additionally, As-IV decreased the amount of autophagosome and expressions of PINK1 and Parkin, but upregulated the expressions of PI3K, p-AKT, and p-mTOR proteins. However, cotreatment with LY294002 diminished the upregulation of PI3K, p-AKT, and p-mTOR induced by As-IV. In the study, we demonstrated that As-IV protected H9c2 cells from H_2_O_2_-induced mitochondrial dysfunction by inhibiting mitophagy, which might be related to the PI3K/AKT/mTOR pathway.

## 1. Introduction

Oxidative stress is the imbalance between the production of reactive oxygen species (ROS) and the capacity of antioxidant defenses, which results in the abnormal accumulation of ROS [1]. A basal level of ROS exerts physiological functions in signaling and stress responsiveness [2]. However, excessive ROS induces protein and lipid peroxidation and also damages DNA and cellular membranes, eventually leading to irreversible cardiomyocyte damage or death [3, 4]. Notably, the mitochondrial electron transport chain is the primary source of ROS in cardiomyocytes, rendering the mitochondria vulnerable to ROS-induced damage [5]. In addition to ATP production, the mitochondria participate in fatty acid oxidation, steroid metabolism, protein quality control, and modulation of calcium homeostasis and redox balance [6–8]. The heart is a highly energy-demanding organ, as such, the mitochondria are abundant in the heart and generate more than two-thirds of cardiac energy [9]. Mitochondrial oxidative damage reduces cardiac energy production, causes mitochondrial calcium overload, disrupts mitochondrial dynamics, promotes the inflammatory response, and induces apoptosis [10]. Consequently, oxidative stress and mitochondrial dysfunction play a significant role in the pathogenesis of cardiovascular diseases, especially heart failure (HF) [11, 12]. It is crucial to maintain mitochondrial homeostasis in cardiomyocytes [13].

Mitophagy, a highly selective form of autophagy, exerts a dual role in mitochondrial homeostasis [14], and under physiological conditions, it reduces mitochondrial ROS accumulation, prevents mitochondrial apoptosis, and promotes the survival of cardiomyocytes by eliminating damaged mitochondria promptly [15]. However, maladaptive mitophagy targets the healthy mitochondria and degrades most of the mitochondria, inducing mitochondrial dysfunction and cardiomyocyte death [16]. Phosphatase and tensin homolog (PTEN)–induced kinase 1 (PINK1)/Parkin-dependent mitophagy is one of the best characterized forms of mitophagy, which is essential for mitochondrial quality control [17]. Under myocardial stress, PINK1/Parkin-dependent mitophagy is significantly upregulated. However, the loss of PINK1 leads to the inhibition of mitophagy, resulting in the accumulation of ROS and inflammation, which ultimately causes cardiomyocyte death [18]. Thus, restoring impaired mitophagy might help alleviate mitochondrial dysfunction, paving a potential strategy for cardiovascular disease therapy.

As the most critical ingredient of traditional Chinese medicine, *Astragalus membranaceus*, Astragaloside IV (As-IV), has diverse pharmacological functions, such as attenuating oxidative stress, suppressing inflammation, and regulating the immune system [19, 20]. In recent years, As-IV has been widely studied for its potential therapeutic benefits in cardiovascular diseases. Accumulating evidence indicates that As-IV has several cardioprotective effects, such as reducing myocardial injury [21], inhibiting myocardial fibrosis [22, 23], improving endothelial dysfunction [24], promoting angiogenesis [25], and regulating cardiac energy metabolism [26]. In our previous study [27], we demonstrated that As-IV attenuates oxidative stress damage and improves left ventricular function via the endothelial nitric oxide synthase (eNOS)/nitric oxide (NO)/cyclic guanosine monophosphate (cGMP) pathway. However, the effects and mechanisms of As-IV on mitochondrial function have not yet been elucidated.

Phosphatidylinositol 3-kinase (PI3K)/protein kinase B(AKT)/mechanistic target of rapamycin (mTOR) is involved in various cellular biological processes, including cell proliferation, apoptosis, and metabolism [28]. Our previous research also revealed that PI3K/AKT was associated with cardiac fibrosis. Moreover, the PI3K/AKT/mTOR pathway is a key regulator of autophagy. Kanno and Hara [29] discovered that rapamycin, an mTOR inhibitor, attenuated doxorubicin-induced cardiomyocyte apoptosis involving the induction of autophagy. Recent studies suggested that the PI3K/AKT/mTOR pathway also serves essential roles in regulating mitochondrial function and mitophagy [30, 31].

As-IV has been extensively studied for its role in regulating the PI3K/AKT/mTOR pathway. It has been reported that As-IV could inhibit idiopathic pulmonary fibrosis by promoting autophagy via the PI3K/AKT/mTOR pathway. Additionally, As-IV exerted proangiogenic effects in myocardial infarction through activating the PI3K/AKT/mTOR pathway [25]. Recent research demonstrated that the combination of Tanshinone IIA and As-IV significantly attenuated atherosclerotic plaque vulnerability in ApoE ^(-/-)^ mice, which was associated with the PI3K/AKT pathway [32]. Moreover, As-IV has been shown to reduce the expression of inflammatory factors by inhibiting the PI3K/AKT/mTOR pathway and optimize the gut microbiota composition in all rats [33]. However, the exact mechanism by which As-IV regulates mitophagy in oxidative stress–induced mitochondrial dysfunction remains unclear.

Therefore, the present study aimed at evaluating the protective effects and mechanism of As-IV against H_2_O_2_-induced mitochondrial dysfunction in H9c2 cells. Based on previous conclusions, we hypothesized that As-IV could inhibit mitophagy via the PI3K/AKT/mTOR signaling pathway, thereby alleviating H_2_O_2_-induced mitochondrial dysfunction in H9c2 cells.

## 2. Methods and Treatment

### 2.1. Cell Culture and Reagents

The rat embryonic ventricular myocardial H9c2 cell line was purchased from OBio Technology (China). The cells were cultured in Dulbecco's modified Eagle's medium (DMEM; Hyclone, United States) containing 10% fetal bovine serum (FBS; Gibco, United States) and penicillin/streptomycin (Gibco, United States) at 37.5°C and under 5% CO_2_. The cells were subcultured or treated when they reached 70%–80% confluency.

Then, 3% hydrogen peroxide (H_2_O_2_) and dimethyl sulfoxide (DMSO) were obtained from Sigma–Aldrich (United States). The Annexin V-FITC Apoptosis Detection Kit I was purchased from BD Bioscience (United States). The specific probe 2⁣′,7⁣′-dichlorofluorescein diacetate (DCFH-DA) and LY294002 were obtained from Beyotime Institute of Biotechnology (China). As-IV with a purity of 99%, and 5,5⁣′,6,6⁣′-tetrachloro-1,1⁣′,3,3⁣′-tetraethylbenzimidazole-carbocyanide iodide (JC-1) was procured from Solarbio Bioengineering (China).

### 2.2. Experimental Design

The oxidative stress model was established by treatment with 200 *μ*M H_2_O_2_ (Sigma, United States) for 2 h. LY294002 was used as a PI3K/AKT pathway inhibitor. The cells were divided into six groups: the control group (treated with DMEM as control), the As-IV group (treated with 100 *μ*M As-IV), the H_2_O_2_ group (treated with 200 *μ*M H_2_O_2_), the As-IV + H_2_O_2_ group (treated with 100 *μ*M As-IV and 200 *μ*M H_2_O_2_), the LY294002 + H_2_O_2_ group (treated with 25 *μ*M LY294002 and 200 *μ*M H_2_O_2_), and the As-IV + LY294002 + H_2_O_2_ group (treated with 100 *μ*M As-IV, 25 *μ*M LY294002 and 200 *μ*M H_2_O_2_). As-IV and LY294002 were solubilized in DMSO to prepare stock solutions that were diluted to the required concentrations in DMEM for subsequent experiments, ensuring the final concentration of DMSO was <0.01%.

### 2.3. Annexin V-FITC and PI Staining

The Annexin V-FITC Apoptosis Detection Kit I was used to examine cell apoptosis by flow cytometry. Then, 1 × 10^5^ cells were cultured in a 6-well plate for 24 h. After different interventions, the cells were washed twice with phosphate-buffered saline (PBS) and digested by trypsinization. Then, the cells were collected by centrifugation at 1000 rpm for 5 min and then resuspended in 1× binding buffer. Subsequently, the cells were incubated with 5 *μ*L of FITC and 5 *μ*L of PI at routine temperature for 5 min in the dark and assessed on a Beckman flow cytometer (United States); the data were analyzed using the CytExpert software.

### 2.4. Detection of ROS

DCFH-DA was used to detect intracellular ROS production. After the indicated treatments, cells were harvested by trypsinization and centrifugation at 1000 rpm for 5 min. Then, the cells were resuspended in 10 *μ*M DCFH-DA for 20 min at 37°C in the dark and analyzed on a flow cytometer. DCFH-DA was excited at 488 nm and emitted at 525 nm.

### 2.5. Malondialdehyde (MDA) and Superoxidase Dismutase (SOD) Release in Culture Medium

The levels of SOD and MDA were measured using the relevant commercial kits from the Institute of Solarbio Bioengineering (China). Then, 1 × 10^5^ cells were seeded in 6-well plates for 24 h. After different treatments, the culture medium was collected for analysis according to the manufacturer's instructions.

### 2.6. Detection of Mitochondrial Membrane Potentials (MMPs)

The fluorescent probe JC-1 was used to assess the changes in MMPs. H9c2 cells were cultured in 6-well plates for 24 h and treated as described above, followed by washes with cold PBS and JC-1 staining at 37°C for 20 min in the dark. After incubation, cells were washed twice with cold PBS. Confocal microscopy (a Zeiss LSM 880 laser microscope) was used to observe the fluorescence images. JC-1 monomers with green fluorescence (excitation 490 nm and emission 525 nm) indicated low MMPs, and JC-1 polymers with red fluorescence (excitation 525 nm and emission 590 nm) indicated high MMPs. The ratios of red/green fluorescence densities were calculated and analyzed using ImageJ software to quantify the changes in the relative MMPs.

### 2.7. Transmission Electron Microscope (TEM) Assays

H9c2 cells were cultured in 6-well plates for 24 h and treated as described above. After treatment, the cells were harvested, washed with PBS, and fixed in 2.5% glutaraldehyde with 0.1 M sodium cacodylate buffer at 4°C for 1 h, followed by fixation in 1% osmium tetroxide at room temperature for 1 h. The samples were dehydrated with a graded series of ethanol, embedded in Epon, sliced into ultrathin sections that were mounted on copper mesh grids, and stained with 1% uranyl acetate and lead citrate. TEM images were captured under HT7800 TEM (Hitachi, Japan).

### 2.8. Western Blot Analysis

H9c2 cells were treated as described above and lysed with radioimmunoprecipitation assay buffer (Solarbio, China) containing protease inhibitors (1 mM PMSF; 1:100) on ice for 45 min. The lysates were clarified by centrifugation at 12,000 rpm, 4°C for 20 min to collect the supernatants to determine the protein concentrations by bicinchoninic acid (BCA) assay (Solarbio, China). An equivalent of 20–30 *μ*g protein was separated by 10% or 15% sodium dodecyl sulfate–polyacrylamide gel electrophoresis (SDS-PAGE) and transferred to the polyvinylidene fluoride (PVDF) membrane. Then, the membranes were blocked in 5% nonfat milk for 2 h at room temperature and probed with primary antibodies against Bax (1:1000, Proteintech, China), Drp1 (1:1000, Abcam), FIS1 (1:1000, Proteintech), OPA1 (1:1000, Proteintech), Mfn2 (1:1000, Proteintech), LC3 (1:1000, Proteintech), P62 (1:1000, Proteintech), PINK1 (1:1000, Proteintech), Parkin (1:1000, Proteintech), PI3K (1:1000, Proteintech), AKT (1:1000, Proteintech), p-AKT (1:1000, Proteintech), mTOR (1:1000, Cell Signaling Technology), and p-mTOR (1:1000, Proteintech) at 4°C overnight. Subsequently, the membranes were incubated with horseradish peroxidase-conjugated secondary antibodies (rabbit, 1:1000, Proteintech or mouse, 1:1000, Proteintech) at room temperature for 1 h. Finally, the immunoreactive bands were visualized by the enhanced chemiluminescence system, and the images were quantitatively analyzed using ImageJ software. GAPDH served as the loading control for whole-cell proteins.

### 2.9. Statistical Analysis

Data were analyzed using SPSS Version 16.0 statistical analysis software, and results were represented as mean ± standard deviation (SD). Statistical significance was determined using one-way analysis of variance (ANOVA). *p* < 0.05, *p* < 0.01, or *p* < 0.001 indicated a statistically significant difference.

## 3. Results

### 3.1. As-IV Reduced the Apoptosis of H_2_O_2_-Induced Injury

**Figure 1:**
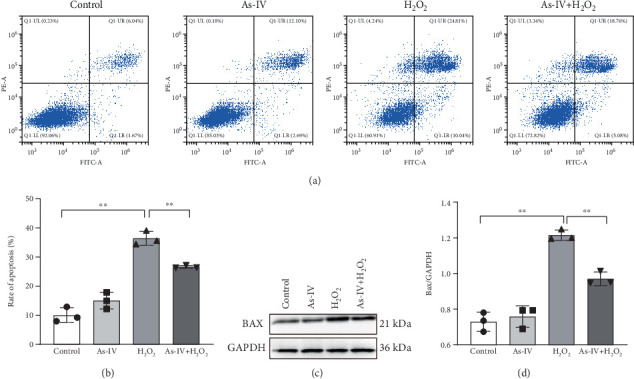
Effects of As-IV on apoptosis in H_2_O_2_-damaged H9c2 cells. (a) Representative images of flow cytometry analysis. (b) Apoptosis rate quantification in different groups. (c) Representative images of Bax expression in H9c2 cells. (d) Protein expression levels were quantified as fold-change relative to GAPDH control. *n* = 3. ⁣^∗∗^*p* < 0.01.

H9c2 cells were exposed to H_2_O_2_ at a concentration of 200 *μ*M for 2 h to develop the oxidative stress injury model. Subsequently, H9c2 cells were pretreated with 100 *μ*M As-IV and then incubated with 200 *μ*M H_2_O_2_ for 2 h to determine the effect of As-IV on apoptosis induced by H_2_O_2_. We measured the proportion of Annexin V-positive cells selected by flow cytometry and assessed the expression of Bax protein to evaluate the apoptosis of cells. The proportion of Annexin V-positive cells was 36.39 ± 2.41% in the H_2_O_2_ group. However, the ratio was significantly reduced to 26.63 ± 0.48% after As-IV treatment (Figure 1a,b). In addition, compared with the H_2_O_2_ group, pretreatment with As-IV significantly decreased the expression of Bax protein (*p* < 0.01) (Figure 1c,d). These results indicated that As-IV reduced apoptosis in H_2_O_2_-induced injury.

### 3.2. As-IV Reduced H_2_O_2_-Induced ROS Generation and Increased Antioxidant Enzyme Activity

**Figure 2:**
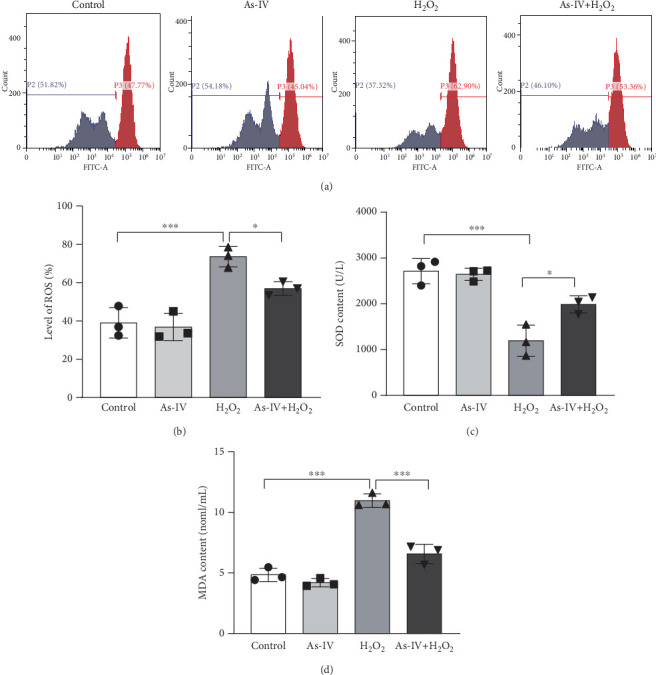
Effect of As-IV on oxidative stress markers in H_2_O_2_-damaged H9c2 cells. (a) Representative images of flow cytometry analysis. (b) Quantification of ROS levels in different experimental groups. (c) SOD content in H9c2 cells. (d) Levels of MDA in H9c2 cells. *n* = 3. ⁣^∗∗∗^*p* < 0.001, ⁣^∗∗^*p* < 0.01, and ⁣^∗^*p* < 0.05.

In order to assess the antioxidant effect of As-IV in H9c2 cells exposure to H_2_O_2_, we detected the level of ROS and MDA and the activity of SOD in different groups. In the H_2_O_2_ group, the level of ROS was 73.53 ± 5.33% and the MDA content was 10.97 ± 0.56 nmol/mL, which were significantly increased (*p* < 0.001, vs. control) (Figures 2a, 2b, and 2d). However, compared to the H_2_O_2_ group, the level of ROS and MDA generation were reduced to 56.93 ± 3.59% and 6.58 ± 0.79 nmol/mL, respectively, in the As-IV with the H_2_O_2_ group (Figures 2a, 2b, 2d). As one of the critical antioxidant enzymes in cells, SOD scavenges excessive ROS. The results in Figure 2b showed that the SOD activity in the H_2_O_2_ model group was significantly lower (1192 ± 341.6 U/L) than that in the control group (2711 ± 274.3 U/L) (*p* < 0.001) (Figure 2c). Compared to the H_2_O_2_ group, the activity of SOD activity was significantly increased to 1985 ± 188.8 U/L when cells were pretreated with As-IV and then incubated with H_2_O_2_ for 2 h (*p* < 0.05), indicating that AS-IV enhanced the activity of the antioxidant enzyme in cells.

### 3.3. As-IV Protected Mitochondrial Function Against H_2_O_2_-Induced Injury

**Figure 3:**
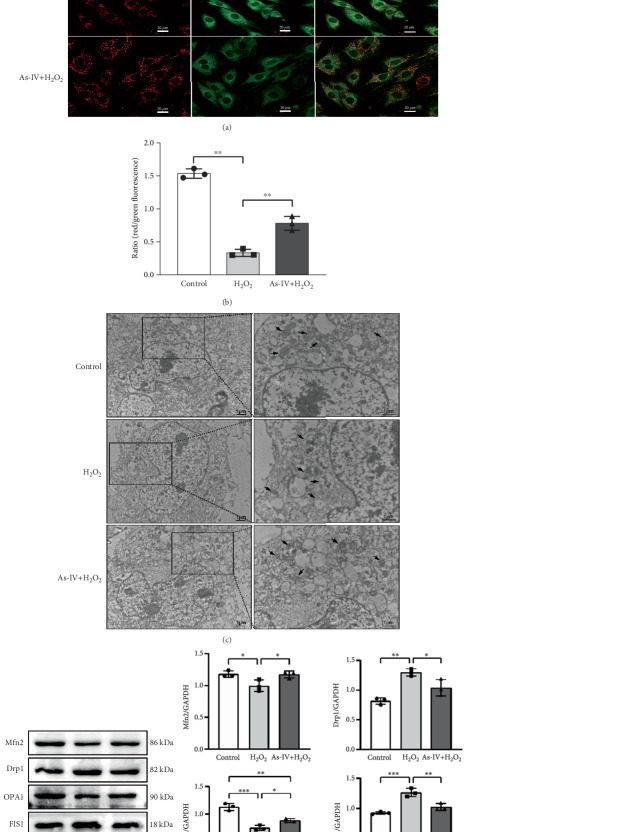
As-IV attenuated H_2_O_2_-induced mitochondrial injury in H9c2 cells. (a) Representative fluorescence microscopy images (scale bar: 20 *μ*m) show that As-IV increases the MMPs in H9c2 cells. JC-1 polymers (red fluorescence) indicate intact MMPs, while JC-1 monomers (green fluorescence) indicate decreased MMPs. (b) Ratio of red/green fluorescence densities was quantified to assess changes in relation to the changes in MMPs. One dot represents the mean fluorescence intensity, which was calculated by selecting a single field of view for each confocal dish and randomly choosing six cells within that field to measure their fluorescence intensity. (c) Representative TEM images showing mitochondrial morphology and autophagic vacuolization in H9c2 cells from different experimental groups (scale bar: 1 *μ*m). (d) Representative images of Mfn2, Drp1, OPA1, and FIS1 expression in H9c2 cells. (e) Protein expression levels were quantified as fold-change relative to GAPDH control. *n* = 3. ⁣^∗∗^*p* < 0.01 and ⁣^∗^*p* < 0.05.

The MMP and balance of mitochondrial fission/fusion are valuable indicators of mitochondrial function. The decline in MMP is an early indicator of mitochondrial damage. To evaluate the effect of As-IV on mitochondrial function, we firstly calculated the change in MMPs. In the control group, the JC-1 probe was detected in the aggregated state, resulting in a high ratio of red fluorescence to green fluorescence.  Figure 3a shows that the exposure of H9c2 cells to H_2_O_2_ reduced the ratio of red to green fluorescence, indicating a decline in MMPs. Compared to the H_2_O_2_ group, As-IV significantly increased the ratio of red to green fluorescence in the H_2_O_2_ + As-IV group, suggesting an increase in MMPs (*p* < 0.01) (Figure 3a,b). In addition, we used TEM to observe the mitochondrial morphology. Damaged mitochondria were detected in H9c2 cells after exposure to 200 *μ*M H_2_O_2_ (Figure 3c). Specifically, the mitochondria were swollen and their cristae were fractured or fuzzy. However, in the As-IV + H_2_O_2_ group, the number of damaged mitochondria was markedly reduced (Figure 3c). Moreover, mitochondrial dynamics were evaluated to assess mitochondrial function. We examined the expression of key proteins involved in mitochondrial fusion and fission. The results are illustrated in Figure 3d,e. H_2_O_2_ injury disrupted the mitochondrial dynamics, manifested as higher expression of Drp1 and FIS1, and lower expression of Mfn2 and OPA1 than the control group in H9c2 cells (*p* < 0.01). However, when H9c2 cells were pretreated with As-IV prior to H_2_O_2_ treatment, the expression of Drp1 was significantly downregulated (*p* < 0.01 vs. the H_2_O_2_ group), and the expression of Mfn2 and OPA1 was upregulated compared to that in the H_2_O_2_ group (*p* < 0.05 vs. the H_2_O_2_ group), which restored the mitochondrial dynamics homeostasis in H_2_O_2_-injured cardiomyocytes. In conclusion, these observations proved that As-IV could partially moderate the decline in MMPs, exert regulatory effects on mitochondrial dynamics, and ameliorate the destruction of mitochondrial structure in H_2_O_2_-injured cardiomyocytes.

### 3.4. As-IV Inhibited Mitophagy During H_2_O_2_-Induced Injury

**Figure 4:**
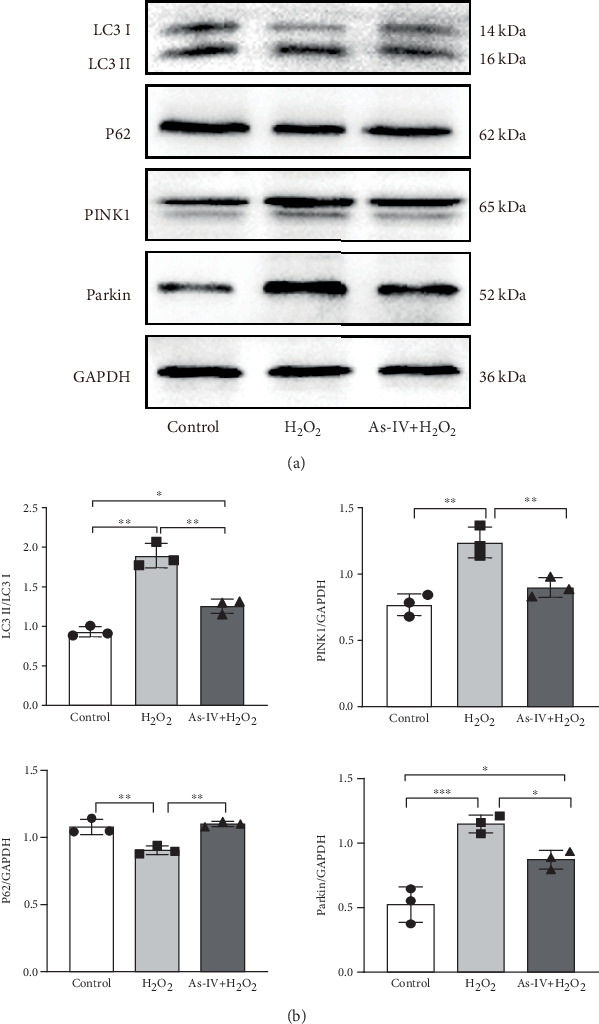
As-IV inhibited mitophagy during H_2_O_2_-induced injury. (a) Representative images of the expression of LC3, P62, PINK1, and Parkin proteins in different groups. (b) Protein expression levels were quantified as fold-change relative to GAPDH control, and the ratio of LC3II/LC3I was calculated. Protein signals as a fold-change relative to GAPDH control and the ratio of LC3II/LC3I are quantified. *n* = 3. ⁣^∗∗∗^*p* < 0.001, ⁣^∗∗^*p* < 0.01, and ⁣^∗^*p* < 0.05.

To further investigate whether As-IV attenuated mitochondrial function by inhibiting mitophagy, we evaluated the autophagosomes and expression of mitophagy-related proteins. As illustrated in Figure 3(c), compared to the control group, the number of autophagosomes was significantly increased in H9c2 cells after exposure to 200 *μ*M H_2_O_2_. Similarly, the ratio of LC3II/LC3I proteins and expression of PINK1 and Parkin proteins were higher, while the expression of P62 was lower in the H_2_O_2_ group than in the control group (*p* < 0.01) (Figure 4), suggesting that H_2_O_2_ overactivated mitophagy in H9c2 cells. However, in the As-IV with H_2_O_2_ group, the number of autophagosomes was reduced markedly (Figure 3(c)). Additionally, compared to the H_2_O_2_ group, the ratio of LC3II/LC3I, PINK1, and Parkin proteins decreased significantly, and P62 proteins increased in the As-IV with H_2_O_2_ group (*p* < 0.01 and *p* < 0.05) (Figure 4). The results indicated that H_2_O_2_-induced oxidative stress overactivated mitophagy, whereas treatment with As-IV significantly inhibited H_2_O_2_-induced mitophagy.

### 3.5. As-IV Activated PI3K/AKT/mTOR Pathway During H_2_O_2_-Induced Injury

**Figure 5:**
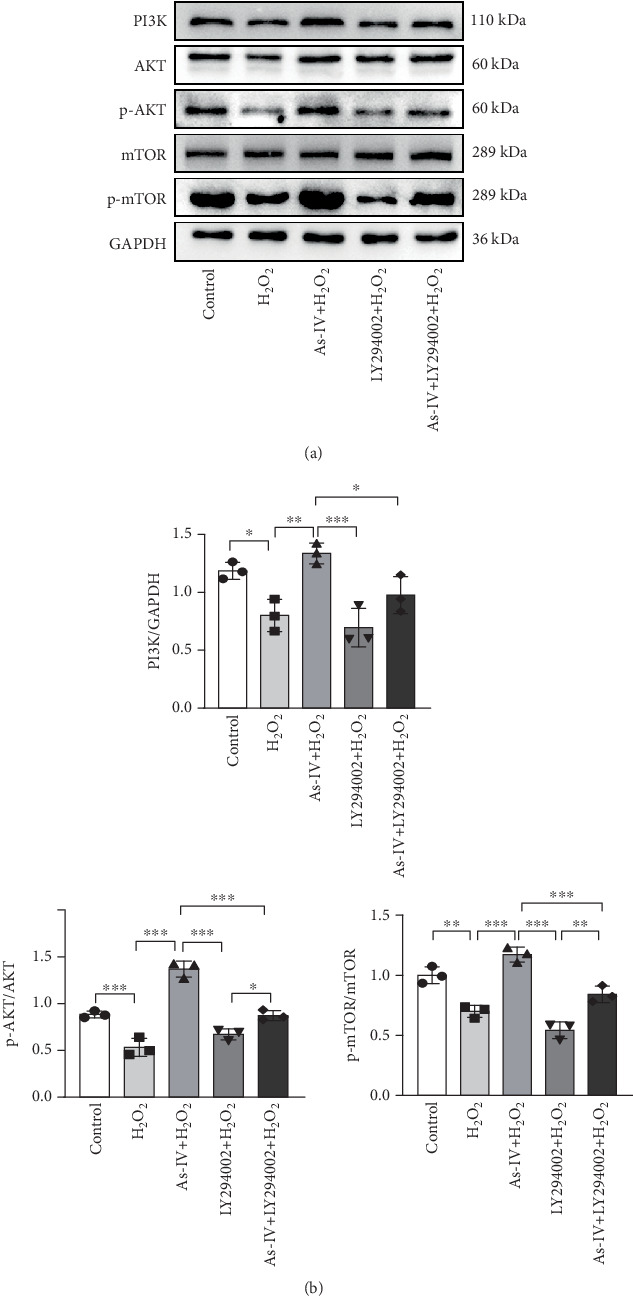
As-IV activated PI3K/AKT/mTOR pathway during H_2_O_2_-induced injury. (a) Representative images showing the expressions of PI3K, AKT, p-AKT, mTOR, and p-mTOR proteins in different groups. (b) Protein expression levels were quantified as fold-change relative to GAPDH control. *n* = 3. ⁣^∗∗∗^*p* < 0.001, ⁣^∗∗^*p* < 0.01, and ⁣^∗^*p* < 0.05.

To investigate the role of the PI3K/AKT/mTOR pathway in As-IV-mediated inhibition of mitophagy, we used LY294002, a PI3K inhibitor, to confirm whether the PI3K/AKT/mTOR pathway was involved in the regulation of As-IV on mitochondrial dysfunction. Accordingly, we examined the expression of PI3K, p-AKT, AKT, mTOR, and p-mTOR in H9c2 cells in different experimental groups. As shown in Figure 5a,b, the expressions of PI3K, p-AKT, and p-mTOR were significantly decreased in the H_2_O_2_ group (*p* < 0.05 vs. control), whereas As-IV treatment significantly upregulated the expressions of PI3K, p-AKT, and p-mTOR proteins (*p* < 0.01 vs. control). Furthermore, treatment with LY294002diminished the upregulation of p-AKT and p-mTOR induced by As-IV (*p* < 0.001 and *p* < 0.05). These results demonstrated that As-IV activated the PI3K/AKT/mTOR pathway during H_2_O_2_-induced injury.

## 4. Discussion

This study investigated the protective effects and underlying mechanisms of As-IV on H_2_O_2_-mediated mitochondrial dysfunction in H9c2 cells. Our results confirmed that As-IV reduced apoptosis, attenuated oxidative stress, and protected H9c2 cells from H_2_O_2_-mediated mitochondrial dysfunction. These protective effects were associated with the inhibition of mitophagy, which might involve the PI3K/AKT/mTOR pathway.

Oxidative stress and mitochondrial dysfunction play significant roles in the pathogenesis of cardiovascular diseases [10, 34]. Excessive ROS production contributes to mitochondrial damage. Consequently, the compromised function of the mitochondria, being the primary source of ROS production, leads to an amplification of oxidative stress, creating a vicious circle [35]. In our oxidative stress model, H9c2 cells exposed to H_2_O_2_ exhibited increased ROS levels, reduced antioxidant enzyme activity, disrupted mitochondrial dynamics, decreased MMP, and altered mitochondrial morphology. These findings are consistent with previous studies indicating that oxidative stress disrupts mitochondrial homeostasis in cardiomyocytes.

Mitochondrial dynamics primarily refer to the continuous processes of mitochondrial fission and fusion, which are essential for maintaining the shape, distribution, and size of the mitochondria [36]. Mitochondrial fission is mediated by Drp1, FIS1, and mitochondrial fission factor (MFF). Mitochondrial fusion involves the merging of the outer and inner mitochondrial membranes. Mfn1 and Mfn2 mediate the fusion of the outer mitochondrial membrane, while inner membrane fusion is solely dependent on OPA1 [37]. Mounting evidence indicates that mitochondrial dynamics are important in cells characterized by high energy demands, notably cardiomyocytes, and an imbalance of mitochondrial dynamics could induce the progression of cardiovascular diseases [38]. Consistent with previous studies, our present work indicated that after cardiomyocyte exposure to oxidative stress, the balance of mitochondrial dynamics was tipped and mitochondrial fission increased, resulting in mitochondrial fragmentation.

Over the past few decades, it has been confirmed that As-IV has antiapoptosis and antioxidant effects [39, 40]. Zhang et al. demonstrated that As-IV could decrease oxidative stress and improve cardiac remodeling by inhibiting the ROS/caspase-1/GSDMD pathway in myocardial infarction models [41]. Additionally, it was reported that As-IV improved cardiac function especially systolic function through alleviating oxidative stress via the Nrf2/HO-1 pathway [42]. Our results corroborated these findings, showing that As-IV enhances intracellular antioxidant defense, restores mitochondrial dynamics, and alleviates H_2_O_2_-induced mitochondrial injury. As-IV mitigated oxidative stress and attenuated apoptosis in cardiomyocytes. The results indicated that As-IV ameliorated H_2_O_2_-metiated mitochondrial damage, characterized by the restoration of mitochondrial dynamics, elevation of MMP, and enhancement of mitochondrial ultrastructure. These results are consistent with previous studies, highlighting the therapeutic potential of As-IV for alleviating oxidative-stress induced mitochondrial dysfunction.

Mitophagy is a pivotal process in mitochondrial quality control. Following MMP depolarization, damaged mitochondria are accurately identified by PINK1, which accumulates on the outer mitochondrial membrane. Subsequently, PINK1 recruits Parkin from the cytosol to the outer mitochondrial membrane, enhancing the activity of Parkin E3 ubiquitin ligase and initiating autophagic flux [43]. We observed that oxidative stress overactivated the PINK1/Parkin-dependent mitophagy. As-IV significantly inhibited excessive mitophagy and improved mitochondrial function. Previous studies have reported that As-IV inhibited mitophagy through PINK1/Parkin signaling in myoblasts [44], consistent with our observations in cardiomyocyte.

Furthermore, we explored the molecular mechanism of As-IV-mediated protective effects on the mitochondria. As well known, the pharmacological effects of As-IV were involved in multiple pathways [45]. Shen et al. found that As-IV attenuated mitochondrial dysfunction and podocyte apoptosis by activating Nrf2-ARE/TFAM signaling [46]. Besides, As-IV could inhibit ferroptosis via the Nrf2/SLC7A11/GPX4 pathway to protect PM2.5-mediated lung injury [47]. It was also found that As-IV reduced adriamycin-induced heart damage and regulated autophagy through activating the PI3K/AKT pathway [48]. The PI3K/AKT signaling pathway was mainly found to be involved in apoptosis or oxidative stress biological processes. As-IV, as an exogenous antioxidant, have a strong relationship with the PI3K pathway. PI3K/AKT/mTOR is a classical signaling pathway related to autophagy, especially since mTOR is a negative regulator of autophagy [49]. Our findings showed that As-IV upregulated the expressions of PI3K, p-AKT, and p-mTOR proteins. While the PI3K inhibitor LY294002 reversed these effects, confirming the involvement of the PI3K/AKT/mTOR pathway in As-IV-mediated mitophagy regulation, the results were in line with the other studies indicating that As-IV modulates autophagy via the PI3K/AKT/mTOR pathway in both in vitro and in vivo models [50].

Nevertheless, the current study has certain limitations. First, while we demonstrated that As-IV regulated the PI3K/AKT/mTOR signaling pathway and mitophagy, other potential mechanisms and direct molecular targets of As-IV remain unexplored. Second, our investigation focused on Parkin-dependent mitophagy, without assessing Parkin-independent pathways. Additionally, the assessment of mitochondrial function involves various dimensions, with mitochondrial bioenergetics and mitochondrial respiration being significant components of mitochondrial functionality. Therefore, comprehensive assessments of mitochondrial function are needed in future studies. Moreover, we used an oxidative stress model in H9c2 cells rather than primary cardiomyocytes or disease-specific models. Future studies should explore the effects of As-IV in primary cardiomyocytes and relevant animal models to confirm its therapeutic potential in cardiovascular diseases. Lastly, further studies are needed to investigate mitophagy and mitochondrial function in the presence of a PI3K inhibitor to better clarify the role of the PI3K/AKT/mTOR pathway in As-IV-mediated mitochondrial protection.

## 5. Conclusion

**Figure 6:**
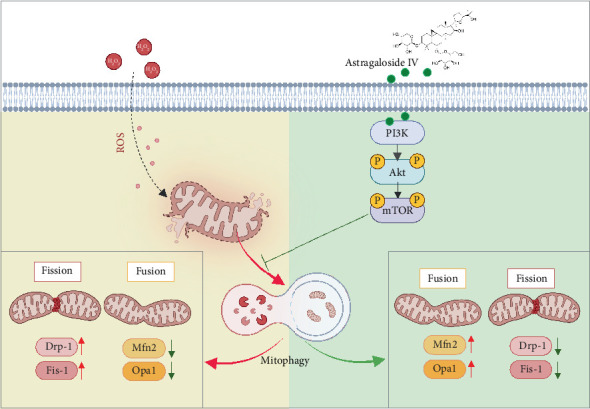
Schematic illustration of the mechanism by which As-IV attenuated mitochondrial dysfunction in H_2_O_2_-induced H9c2 cell injury. H_2_O_2_ induced oxidative stress, resulting in an imbalance of mitochondrial dynamics. As-IV regulated mitochondrial dynamics by inhibiting mitophagy, which might be associated with the PI3K/AKT/mTOR pathway.

In conclusion, our study indicated that As-IV protected H9c2 cells from H_2_O_2_-induced mitochondrial dysfunction by inhibiting mitophagy, which might be closely associated with upregulation of the PI3K/AKT/mTOR pathway (Figure 6). The results provided evidence that As-IV could be a therapeutic target in cardiovascular diseases. As-IV could potentially serve as a strategy to prevent oxidative stress-induced cardiomyocyte mitochondrial dysfunction.

## Data Availability

The data used to support the findings of this study are available from the corresponding author upon request.
